# Cross-Talk between PPARs and the Partners of RXR: A Molecular Perspective

**DOI:** 10.1155/2009/925309

**Published:** 2009-12-20

**Authors:** Lap Shu Alan Chan, Richard A. Wells

**Affiliations:** ^1^Department of Medical Biophysics, University of Toronto, Toronto, ON, Canada M5G 2M9; ^2^Discipline of Molecular and Cellular Biology, J. Douglas Crashley Myelodysplastic Syndrome Laboratory, Sunnybrook Research Institute, 2075 Bayview Avenue, T2-058, Toronto, ON, Canada M4N 3M5; ^3^Department of Medical Oncology, Crashley Myelodysplastic Syndromes Research Laboratory, Odette Cancer Centre, Sunnybrook Health Sciences Centre, Toronto, ON, Canada M4N 3M5; ^4^Department of Medicine, University of Toronto, Toronto, ON, Canada M4G 2C4

## Abstract

The PPARs are integral parts of the RXR-dependent signaling networks. Many other nuclear receptor subfamily 1 members also require RXR as their obligatory heterodimerization partner and they are often co-expressed in any given tissue. Therefore, the PPARs often complete with other RXR-dependent nuclear receptors and this competition has important biological implications. Thorough understanding of this cross-talk at the molecular level is crucial to determine the detailed functional roles of the PPARs. At the level of DNA binding, most RXR heterodimers bind selectively to the well-known “DR1 to 5” DNA response elements. As a result, many heterodimers share the same DR element and must complete with each other for DNA binding. At the level of heterodimerization, the partners of RXR share the same RXR dimerization interface. As a result, individual nuclear receptors must complete with each other for RXR to form functional heterodimers. Cross-talk through DNA binding and RXR heterodimerization present challenges to the study of these nuclear receptors that cannot be adequately addressed by current experimental approaches. Novel tools, such as engineered nuclear receptors with altered dimerization properties, are currently being developed. These tools will enable future studies to dissect specific RXR heterodimers and their signaling pathways.

## 1. Introduction

The PPARs are one of the most studied RXR heterodimerization partners.Over 1000 papers have been published on the PPARs since their cloning in 1990 [1].Although some evidence suggests that the PPARs can form homodimers and bind to DNA response elements such as the Pal3 motif [2], it is widely accepted that the PPARs must heterodimerize with RXR to carry out most of their functions. Therefore, like other RXR's partners, the PPARs are integral parts of the RXR-dependent signaling network—RXR is also the obligatory heterodimerization partner of at least twenty mammalian nuclear receptors (NRs) [3–10]. Since studies in mouse have shown that multiple RXR's partners are often coexpressed in any given tissue [11–16] (also see the Nuclear Receptor Signaling Atlas (NURSA) [17]), it appears that cross-talk between these partners is a common phenomenon. Cross-talk between these partners is complex and presents a unique challenge to researchers who are trying to understand the function of individual NR.

This paper will focus on the basis of the cross-talks between RXR's partners from a molecular biology perspective, where these NRs compete against each other for DNA binding and RXR heterodimerization. In addition, this paper will discuss the challenges faced by investigators using current experimental approaches to dissect and understand the functional role of individual partners of RXR.

## 2. How Do RXR Heterodimers Bind to Their DNA Response Elements?

**Figure 1:**
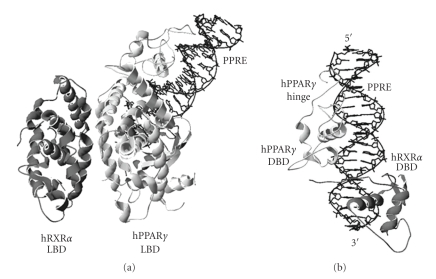
(a) Ribbon drawing of the human RXR*α*/human PPAR*γ* heterodimer on PPRE (PBD 3E00). (b) Ribbon drawing of the heterodimer/DNA complex showing only the PPRE and the DBDs of RXR*α* and PPAR*γ*. The RXR and PPAR monomers are colored in dark and light grey, respectively. The PPRE is colored in black; the 5′ and 3′ ends are also labeled.

Most RXR/partner heterodimers recognize and bind to direct repeat (DR) DNA sequences as their response element [18–23]. The consensus DR sequence consists of two direct repeat half-sites separated by a number of nucleotides (5′-AGGTCA *n*(*x*) AGGTCA-3′). A DR separated by a single nucleotide is referred to as DNA response element direct repeat 1 (DR1). In addition, the sequence of the actual 5′-AGGTCA-3′ half-site varies among different response elements. The RXR*α*/PPAR*γ* heterodimer preferentially binds to the DR1 PPAR response element (PPRE) [24], and the crystal structure of this heterodimer/DNA complex is recently reported (Figure 1) [25]. During DNA binding, the heterodimer is arranged such that the DNA binding domain (DBD) from each monomer occupies one 5′-AGGTCA-3′ half-site. Most heterodimers are selective toward DR sequences with one to five nucleotides spacing (i.e., DR1 to DR5). At the molecular level, addition or subtraction of a single base pair between the half-sites imposes a separation of 3.4 Å and a rotation of 36° between the two half-sites [19]. Since a DBD is only slightly longer than one half-site, the structural conformations required for the heterodimers vary greatly for different DRs. Heterodimerization of the DBDs on their correct DR elements helps define the structural conformation of the heterodimer, which in turn stabilizes the protein/DNA complex. It is also worth noting that the RXR DBD is flexible and undergoes structural changes to accommodate the DBDs from different partners.

There are two additional consequences to these DNA binding properties. First, unlike the inverted repeat DNA sequence used by other NRs, the DR sequence is asymmetrical and can only be read correctly from one direction [26–28]. This restricts the protein complex to bind to the DNA in only one orientation, in contrast to inverted repeats or everted repeats, which can interact with their cognate NR complex in both 5′ and 3′ orientations. Consequently, the protein complex exerts its effect in a unidirectional manner. Second, unlike homodimers, heterodimers can bind to response elements with RXR occupying either the 5′ upstream or 3′ downstream half-site [29–32]. This difference in polarity effectively doubles the number of possible RXR/partner heterodimer combinations, even though not all combinations are capable of DNA binding. In addition, different RXR polarity can result in completely different gene regulatory responses. For instance, although the RXR-RAR heterodimer binds to both DR1 and DR5, the RXR monomer occupies the 3′ half-site of DR1 and the 5′ half-site for DR5 [30]. This allows ligand-dependent activation for RXR-RAR heterodimer on DR5, but not on DR1. Hence, different genes can be regulated in different manner by a given heterodimer and ligand. The crystal structure of RXR*α*/PPAR*γ* heterodimer on the PPRE reveals that PPAR*γ* resides upstream of RXR*α*, resembling the organization of RXR/RAR on DR1 [25] (Figure 1). However, it is unclear whether PPAR*γ* can also reside downstream of RXR*α* and exert different activation properties.

Since there are more than forty heterodimer combinations and only five DR response elements, many heterodimers share the same DR element (Table 1) [19]. This implies that different heterodimers must compete for DNA binding. A well-known example is that the RXR homodimer and several other RXR heterodimers can recognize and bind to the DR1 element [29]. Subsequently, cross-talk becomes possible among different heterodimers and the signaling pathways they represent. Several mechanisms exist to control the relative affinity of these dimers to DR1 [33]. First, the expression level of NRs varies among different cell types; thus, protein abundance may determine DNA binding [11]. Second, the precise sequence of the DR element influences the binding specificity of different dimers. For instance, the PPREs have a well conserved AAACT extension sequence located upstream of the DR site [34]. The crystal structure of RXR*α*/PPAR*γ* heterodimer revealed that the hinge region of PPAR*γ* can recognize this AAACT element and allow specific PPREs binding [25]. Nevertheless, the specificity of RXR/PPAR heterodimer to PPRE does not necessarily preclude DNA binding by other RXR heterodimers that also recognize the DR1 element.

## 3. How Do RXR and Its Partners Form Heterodimers?

Although RXR and its partners may perform certain functions as monomers or homodimers, the heterodimers are responsible for most gene regulatory activities. Therefore, it is reasonable to regard the heterodimer as the basic functional unit of the signaling network, rather than viewing RXR and its partner monomer as separate units. Numerous studies have been conducted to investigate the heterodimerization properties of RXR and its partners using X-ray crystallography and mutational analysis of the amino acid sequence. Although both the DBD and the ligand binding domain (LBD) are involved in dimerization, the LBD is more important due to its much larger and stronger dimerization interface [35, 36]. Therefore, studies on RXR/partner heterodimerization have mainly focused on the LBD.

### 3.1. The Structure of RXR Homo/Heterodimer

**Figure 2:**
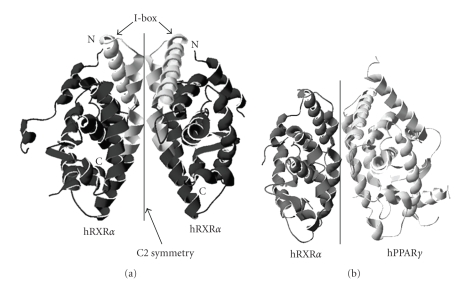
(a) Ribbon drawing of the human RXR*α* LBD homodimer (PBD 1MZN). The RXR monomers are colored in black. The N and C termini are labeled “N” and “C”, respectively. Rotation of 180° on the Z-axis of the homodimer results in a geometry equivalent of the starting geometry (i.e., C2 symmetry). The I-boxes are colored in light grey. (b) Ribbon drawing of the hRXR*α*/hPPAR*γ* LBD heterodimers (PDB 3E00, also see Figure 1). The RXR and PPAR monomers are colored in dark and light grey, respectively.

The RXR LBD homodimer (Figure 2(a)) was the first RXR dimer to be resolved by X-ray crystallography [36]. Each RXR monomer consists of twelve *α*-helices (H1 to H12) and two short *β*-strands (s1 and s2), which are organized in three layers to form an antiparallel “*α*-helical sandwich” [37]. The homodimer has twofold symmetry and forms a rotationally symmetric dimer. The dimer interface consists of amino acid residues that are arranged as a hydrophobic cluster surrounded by charged and polar residues. These residues interact with each other to stabilize the homodimer and therefore are essential for dimerization. The structural arrangement of the PPAR LBD resembles that of the RXR LBD, except the PPAR LBD has one extra *α*-helice and two extra *β*-strands [38]. The crystal structures of several RXR/partner LBD heterodimers have been resolved since the publication of the RXR LBD homodimer, including RXR/RAR [39, 40], RXR/CAR [41, 42], RXR/LXR [43, 44], and RXR/PPAR [25, 45–47]. These heterodimers share the same global structure with the RXR LBD homodimer. Nevertheless, individual heterodimers have specific configurations, which differ from the RXR homodimer prototype. These differences include deviation of a monomer from the symmetry axis, the exact area that the monomers contribute to the dimer interface, and the rearrangement of interactions between amino acid residues from the monomers. For instance, the monomers of the RXR*α*/PPAR*γ* LBD heterodimer (Figure 2(b)) [25] deviate about 10° from the C2 symmetry, which leads to increased surface contact area between the monomers and enhances the stability of the heterodimer [45].

Since each heterodimer possesses different heterodimerization interface, it is reasonable to conjecture that some partners can form more stable heterodimers with RXR than can others, even though the relative stability of each heterodimer is yet to be fully established. It has been suggested that the quantity of RXR available for heterodimerization is limited and under strict control [48, 49]. As a result, cross-talk between RXR's partners can be achieved via RXR sequestration. For instance, the presence of excess LXR or its ligands reduces DNA binding of PPAR*α*/RXR*α* to PPRE [50], which inhibits PPAR signaling and suppresses transcription of lipid degradation genes. Conversely, excess of PPAR*α* and its ligands suppresses the sterol regulatory element-binding protein-1c promoter that contains two LXR response elements [51]. This inhibition can be relieved by the addition of RXR*α*, suggesting that competition for and sequestration of RXR is a contributor to cross-talk between the LXR and PPAR signaling pathways. Other examples of cross-talks between RXR's partners include PPAR with COUP-TF [52], TR with LXR [53], TR with VDR [54], and multiple partners (CAR, PXR, LXR, FXR, and PPAR) on the expression of P450 enzymes [55].

### 3.2. Amino Acid Residues that Are Important in Dimerization

**Figure 3:**
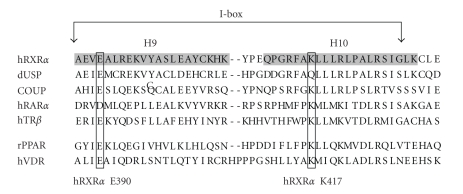
Amino acid sequences of the I-box region of RXR*α*, USP, COUP, RAR*α*, TR*β*, PPAR, and VDR. The I-box lies within Helix 9 (H9) and helix 10 (H10) (shaded) and has been shown to be essential to dimerization. The amino acid residues equivalent to hRXR*α* E390 and K417 are also marked.

The dimer interface holds the key to dimerization, and amino acid residues within this interface are especially important. Early studies showed that deletion of the RXR LBD from amino acid position 443 to the C-terminal end does not disrupt dimerization [56], while additional deletion to position 433 disrupts RXR homodimerization but not heterodimerization with other NRs. Further deletion to position 413 abolishes all dimerization activities. These observations suggested that a short region of RXR 413-443 is required for dimerization, and the region 413-433 is particularly important to heterodimerization. Subsequent analysis by the Evans' group redefined the heterodimerization region as 387-429 and termed this region the “I-box” [57] (Figures 2 and 3). As observed in the crystal structure, the I-box lies within helices H9-H10, which is at the center of the dimer interface. RXR and all of its known partners possess the I-box, and a number of highly conserved amino acid residues are found in this region. Interestingly, RAR acquires the heterodimerization properties of RXR when its I-box is replaced by that of the RXR, and vice versa [57].

Mutational analysis of RXR homo- and heterodimerization has also utilized amino acid mutations through site directed mutagenesis. The results indicated that combination of mutations at the I-box disrupt dimerization, presumably through steric hindrance or charge confliction within the dimer interface. For instance, the Pfahl's group has reported that simultaneous mutations of L418R, L419S, and L422Q can completely abolish RXR homo- and heterodimerization [56]. In addition, other mutations can alter heterodimerization of RXR with certain partners. These mutations include RXR A416D or R421L, which specifically disrupt the formation of the RXR/TR heterodimer, and RXR A416K, which disrupts RXR/RAR and RXR/TR [58]. Likewise, the I-box of the partner of RXR is equally vital to heterodimerization, such as VDR K382E (equivalent to human RXR*α* K417) cannot form heterodimers with RXR [59]. The I-box may also hold the key that determine RXR homo- and heterodimerization. The Gronemeyer's group has reported that a RXR mutant (Y402A) is able to provide additional stabilization to the RXR homodimer interface, making the RXR mutant unavailable for heterodimerization [35]. Overall, these studies identify the I-box as a crucial element for RXR heterodimerization, and mutations at this region can alter heterodimerization properties.

## 4. Studies on the RXR-Dependent Signaling Network and Its Individual Pathways

A number of experimental approaches have been employed to study the function of RXR and its partners, including targeted gene disruption, naturally occurring NR mutants, and heterodimer specific ligand stimulation. Studies using these approaches have contributed greatly to the understanding of the RXR dependent signaling network. However, there are limitations and it remains difficult to address the functional roles of individual heterodimers in isolation.

### 4.1. Targeted Gene Disruption

Gene knockout models have been extensively used to characterize the physiological role of RXRs. RXR*α* knockout (−/−) in mouse is embryonic lethal in mid-gestation because of a noncell autonomous defect in the development of the ventricular myocardium [60, 61]. It is possible to bypass the embryonic lethality using tissue specific conditional knockout [61]. Studies based on this system suggested that RXR*α* has an essential role in multiple pathways including glutathione homeostasis and detoxification of xenobiotics [62], the lifespan and regenerative capacity of hepatocytes [63], proliferation and differentiation of epidermal keratinocytes [64, 65], and development of prostatic intraepithelial neoplasia [66]. Knockout of PPAR*γ* in mouse has established its involvement in the development placental, cardiac, and adipose tissue [67–69], while RARs are involved in spermatogenesis and fetal development [70, 71]. Observations from these gene disruption models have helped to establish the physiological roles of RXR and its partners. However, these models cannot clearly define the exact functions of individual heterodimers because of the unique relationship between RXR and its partners. First, genes from a particular signaling pathway can be coregulated by one or more pathways. For instance, disruption of one RAR isoforms is often compensated by the other two RAR isoforms [70, 71]. This implies that the remaining intact pathways can mask the effect of the knockout by maintaining certain gene regulatory activities normally handled by the absent partner. Second, the absence of a partner through targeted gene disruption increases the availability of RXR and cofactors. Consequently, these excess proteins can enhance the activities of other intact heterodimers and their subsequent signaling pathways.

### 4.2. Heterodimer Specific Ligand Stimulation

Ligand-dependent activities of RXR heterodimers are of major interest in NR research due to their physiological relevance and potential application in medicine. For instance, thiazolidinediones (TZDs) activate PPAR*γ* in brown and white adipocyte, and induce the transcription of PGC-1 alpha. PGC-1 alpha is involved in the control of mitochondrial biogenesis and has been linked to insulin sensitization [72]. Other pharmacological compounds such as WY-14643[73], L-165041 [74], and GW-7845 [75] are specific ligands for *α*, *δ*, and *γ* isoforms, respectively. Bisphenol diglycidyl ether (BADGE) [76] and LG100641 [77] are PPAR*γ* selective antagonists, while nonsteroidal anti-inflammatory drugs (NSAIDs) can block PPAR*δ* specifically [78]. Nevertheless, a number of heterodimer-specific ligands exert effects independent of their receptor. For instance, PPAR*γ* specific ligands troglitazone and 15-deoxy-prostaglandin J2 inhibit growth of prostate and bladder carcinoma cell lines. However, this effect is not blocked by a PPAR*γ* antagonist [79]. This observation leads to the conclusion that the particular growth inhibition effects of both agents are mediated through PPAR*γ* independent mechanisms [79, 80]. Receptor independent effects have also been reported for RAR ligands [81]. Hence, even though these effects may be essential to medical and pharmaceutical applications, they do not contribute to the RXR dependent signaling network and may create confusion in analyzing the functions of individual heterodimers. Furthermore, the use of ligands cannot address the functional role of apo-heterodimers, which binds to their respective response element even in the absence of ligand.

### 4.3. A New Approach to Dissect Individual RXR Signaling Pathway

**Figure 4:**
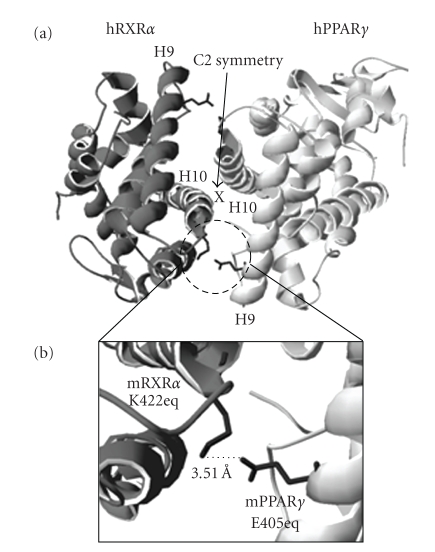
(a) Ribbon drawing of hRXR*α*/hPPAR*γ* LBD heterodimer (PDB 3E00, also see Figure 2). (b) The detail view of the equivalent amino acid sidechains of mouse RXR*α* K422 and mouse PPAR*γ* E405. The shortest distance between the negative (side chain carboxyl group of E405) and positive (side chain amino group of K422) charges is also displayed.

The research of RXR dependent signaling networks requires the precise characterization of individual signaling pathways. However, current experimental approaches are insufficient to achieve this objective due to the unique properties of the signaling network. A novel experimental system consisting of engineered RXR and partners with controllable heterodimerization specificity would be helpful to complement the existing approaches and to circumvent their limitations. Our laboratory has created and tested an engineered RXR/PPAR heterodimer consist of mouse RXR*α* K422E and mouse PPAR*γ* E405K mutants [82]. The creation of these mutants is based on the proposed salt-bridge between RXR*α* K422 and PPAR*γ* E405 (Figure 4). In addition, PPAR*γ* E405 and RXR*α* K422 correspond to human RXR*α* E390 and K417, which are highly conserved among RXR and its partners in mammalian species (Figure 3). Since this salt-bridge is located within the heterodimer interface, we postulate that this salt-bridge may have a role in heterodimerization. We also hypothesized that reversing the polarity of the side chains of these amino acids may alter heterodimerization specificity but the salt-bridge will be kept intact. Indeed, our observations suggest that the mutant pair is able to form a heterodimer. Although PPAR*γ* E405K can form a heterodimer with wild-type RXR*α*, RXR*α* K422E is not able to heterodimerize with wild-type PPAR*γ* (Table 2). In addition, ligand response of the PPAR*γ* E405K mutant is comparable to that of the wild-type PPAR*γ*, suggesting that the general structure of this mutant is preserved. The restricted heterodimerization capacity of the RXR*α* K422E mutant is especially exciting, since the salt-bridge between RXR*α* K422 and PPAR*γ* E405 (Figure 4(b)) is predicted to exist in other RXR's partners based on the crystal structures of the LBD heterodimers (RXR/RAR [39, 40], RXR/CAR [41, 42], RXR/LXR [43, 44], and RXR/PPAR [45–47]). We are currently conducting experiments to determine if the restriction on heterodimerization of RXR*α* K422E is also applicable to other NR partners, and if mutants of these partners (equivalent to PPAR*γ* E405K) can restore heterodimerization with RXR*α* K422E. If true, our approach will permit rescue, in the context of RXR knockout, of specific NR pathway either by knock-in or by in vitro delivery of dimerization restricted NR pairs. Expression of RXR*α* K422E and PPAR*γ* E405K mutants in RXR*α* −/− cells could, for instance, be used to restore specifically the functions mediated by RXR*α*/PPAR*γ* heterodimer, thus identifying the contribution of this particular heterodimer to the whole phenotype. Hence, it maybe possible to dissect the RXR-dependent signaling pathway in a precise manner using dimerization-restricted NR pairs. 

In summary, more than twenty RXR heterodimers share only five common DNA response elements. Although some of these DNA response elements have features that favor binding by specific heterodimer, direct competition for DNA binding is a common phenomenon for these heterodimers. Competition for RXR among the partners of RXR is also intense due to the expression of multiple NRs in the same cell and the limited availability of RXR. There is evidence suggesting that the relative expression level of different NRs and RXR dictates the activity of individual NR pathways. Hence, direct competition for DNA binding and heterodimerization has significant roles in the cross-talk between PPARs and other RXR's partners. These unique properties present challenges to the study of these partners. A novel experimental approach is currently being developed to alter the dimerization properties of selected heterodimers, which will allow future studies to dissect a specific RXR heterodimer and its signaling pathway from the rest of the RXR-dependent signaling network.

## Figures and Tables

**Table 1:** DR element binding properties of RXR homodimers and heterodimers.

DR element	RXR homo/heterodimer
DR1	RXR- RXR, RAR, PPAR, COUP, HNF4
DR2	RXR-PPAR, RAR
DR3	RXR-VDR
DR4	RXR-TR, LXR, CAR
DR5	RXR-RAR, NGFI-B

**Table 2:** Altered heterodimerization properties of the RXR and PPAR charge-reversal mutants.

RXR	PPAR	Heterodimer?
Wide-type	Wide-type	YES
Wide-type	E405K	YES
K422E	Wide-type	NO
K422E	E405K	YES
